# Impact of obstructive sleep apnea on lung volumes and mechanical properties of the respiratory system in overweight and obese individuals

**DOI:** 10.1186/s12890-015-0063-6

**Published:** 2015-07-25

**Authors:** Arikin Abdeyrim, Yongping Zhang, Nanfang Li, Minghua Zhao, Yinchun Wang, Xiaoguang Yao, Youledusi Keyoumu, Ting Yin

**Affiliations:** Postgraduate college of Xinjiang Medical University, Urumqi, China; The People’s Hospital of Xinjiang Uygur Autonomous Region, Urumqi, 830001 China; Department of Pulmonary function test, The People’s Hospital of Xinjiang Uygur Autonomous Region, Urumqi, China; Laboratory of sleep study, The People’s Hospital of Xinjiang Uygur Autonomous Region, Urumqi, China; The Center of Hypertension of the People’s Hospital of Xinjiang Uygur Autonomous Region, Urumqi, China; Department of Otorhinolaryngology Head and Neck Surgery, The People’s Hospital of Xinjiang Uygur Autonomous Region, Urumqi, China

**Keywords:** Lung volume measurements, Functional residual capacity, Elastic properties of lung, Lung elastance, Obstructive sleep apneas

## Abstract

**Background:**

Even through narrowing of the upper-airway plays an important role in the generation of obstructive sleep apnea (OSA), the peripheral airways is implicated in pre-obese and obese OSA patients, as a result of decreased lung volume and increased lung elastic recoil pressure, which, in turn, may aggravate upper-airway collapsibility.

**Methods:**

A total of 263 male (*n* = 193) and female (*n* = 70) subjects who were obese to various degrees without a history of lung diseases and an expiratory flow limitation, but troubled with snoring or suspicion of OSA were included in this cross-sectional study. According to nocturnal-polysomnography the subjects were distributed into OSA and non-OSA groups, and were further sub-grouped by gender because of differences between males and females, in term of, lung volume size, airway resistance, and the prevalence of OSA among genders. Lung volume and respiratory mechanical properties at different-frequencies were evaluated by plethysmograph and an impulse oscillation system, respectively.

**Results:**

Functional residual capacity (FRC) and expiratory reserve volume were significantly decreased in the OSA group compared to the non-OSA group among males and females. As weight and BMI in males in the OSA group were greater than in the non-OSA group (90 ± 14.8 kg vs. 82 ± 10.4 kg, *p <* 0.001; 30.5 ± 4.2 kg/m^2^ vs. 28.0 ± 3.0 kg/m^2^, *p <* 0.001), multiple regression analysis was required to adjust for BMI or weight and demonstrated that these lung volumes decreases were independent from BMI and associated with the severity of OSA. This result was further confirmed by the female cohort. Significant increases in total respiratory resistance and decreases in respiratory conductance (Grs) were observed with increasing severity of OSA, as defined by the apnea-hypopnea index (AHI) in both genders. The specific Grs (sGrs) stayed relatively constant between the two groups in woman, and there was only a weak association between AHI and sGrs among man. Multiple-stepwise-regression showed that reactance at 5 Hz was highly correlated with AHI in males and females or hypopnea index in females, independently-highly correlated with peripheral-airway resistance and significantly associated with decreasing FRC.

**Conclusions:**

Total respiratory resistance and peripheral airway resistance significantly increase, and its inverse Grs decrease, in obese patients with OSA in comparison with those without OSA, and are independently associated with OSA severity. These results might be attributed to the abnormally increased lung elasticity recoil pressure on exhalation, due to increase in lung elasticity and decreased lung volume in obese OSA.

**Electronic supplementary material:**

The online version of this article (doi:10.1186/s12890-015-0063-6) contains supplementary material, which is available to authorized users.

## Background

Obstructive sleep apnea (OSA) is a common condition that is often associated with central obesity [1]. During sleep, maintenance of upper airway patency is a primary physiologic goal, failure of which causes OSA and its sequelae [2], with associated cardio-cerebrovascular complications [3, 4].

Changes in lung volume are well known to affect pharyngeal airway size and stiffness, through thorax caudal traction on the trachea (Ttx) and may predominantly reflect Ttx effects [5, 6], even though the anatomy narrowing of the upper airway plays an important role in the pathogenesis of OSA. There have been many studies examining the effects of lung volume on collapsibility of the human pharynx that have shown a similar response: using negative extrathoracic pressure (NETP) to elevate lung volume above functional residual capacity (FRC) in OSA and normal subjects during wakefulness or sleeping, is accompanied by improvement in pharyngeal collapsibility [7–11], and decrease in pharyngeal resistance due to increases in pharyngeal size [12]. These phenomena appear to be more pronounced in obese patients with OSA possibly because they usually respire with low lung volume. Therefore, obese OSA patients would obtain therapeutic benefits: inflation and maintenance of lung volume above FRC or end-expiratory lung volume (EELV) caused by NETP, or continuous positive airway pressure (CPAP) produces marked decreases in the apnea-hypopnea index (AHI) and in the magnitude of “holding pressure” (CPAP is required to eliminate upper airway flow limitation) [9–11]. These studies appear to suggest involvement of lung volume, in terms of FRC or EELV, in the pathogenesis of OSA [7, 8].

A recent animal study demonstrated that inspiratory resistive breathing (IRB), similar to upper airway obstructed breathing causes significantly increased lung elasticity measured by low-frequency forced oscillation technique (FOT) and downward shift of the pressure-volume curve; as IRB generates large swings in intrathoracic pressures and triggers lung inflammation [13]. It has been shown by Van De Graff in anesthetized dogs that swings in intrathoracic pressure determine the extent of Ttx, which act independently to either draw the trachea into or push the trachea out of the thorax and that reciprocal movements of the trachea are independent of upper airway muscle activity determining alterations in upper airway patency [5, 14]. Accordingly, the suggestions of those studies were that lung elasticity properties significantly influence generation of the Ttx, and that lung volume *per se*, would not determine the mechanical influence of the thorax on the upper airway [14]. Onal reported daytime measurements of airway conductance (Gaw) and the reciprocal of airway resistance (Raw), and predicted FRC were inversely associated, to a strong degree, with severity of OSA as defined by AHI in OSA patients without any obstructive lung disease, and suggested that decreased lung volume and increased Raw contribute to the severity of OSA [15]; Zerah [16] clearly demonstrated in obese subjects that respiratory system resistance (Rrs) measured by FOT was approximately equal to Raw, and the two parameters increased significantly with the degrees of obesity resulting from the reduction in lung volume. Subsequently, Zerah [17] analyzed Rrs data obtained in 170 obese OSA patients, back-extrapolated the regression line to 0 Hz, to obtain the total Rrs and its inverse—respiratory conductance (Grs); because Rrs at lower frequencies (4–16 Hz) on FOT was subjected to linear regression analysis over the 4 to 16 Hz frequency range in obese subjects with and without OSA. The study observed that significant increase in the Rrs, and decreases in the Grs as well as in the specific Grs (sGrs: the ratio of Grs over FRC) were independently associated with OSA severity defined by AHI [17, 18]. The ratio of Gaw over FRC and Gaw were used to reflect a function of the lung elasticity recoil forces, which is an important mechanical property of the respiratory system, as well as determining Raw, and is also sensitive to changes of FRC or EELV [16]. In fact lung volume is determined by the balance of the elastic recoil forces of lungs (inward recoil) and chest wall (outward recoil) [19]. From such evidence, we can imagine that Rrs increases and its inverse—Grs decreases more in obese OSA patients than those without OSA may result from increased lung elasticity recoil pressure as well as decreased in lung volume. Although, the studies mentioned previously appear to indicate to us that lung elasticity would be decreased in OSA patients. However, we can expect, despite the paradoxical evidence, that a vicious cycle exists between OSA and lung elasticity properties, which we feel needs further study.

The impulse oscillation system (IOS) is a type of FOT that has been progressively developed for clinical use over the years, as it was thought to provide information related to lung elasticity properties and Rrs during tidal breathing [20–22]. Systematically assessed lung volumes, and respiratory mechanical properties of OSA patients using IOS may provide new insights into the underlying pathophysiology of OSA.

The aim of this study was to investigate the effect of OSA on lung volumes and mechanical properties of the respiratory system without the influence of body mass index (BMI) differences.

## Methods

### Subjects

In total 290 consecutive subjects (207 males, 83 females) who were obese to various levels without a medical history of lung diseases but who were troubled with snoring or suspicion of OSA were eligible for this cross-sectional study from April 2013 to April 2014. The classification of being pre-obese (overweight) or obese was according to the Chinese criteria [23]: Subjects with a BMI of 24–27 kg/m^2^ were classified as pre-obese; subjects with a BMI of 27.1–40 kg/m^2^ and over 40 kg/m^2^ were classified as obese and morbidly obese, respectively.

The exclusion criteria were patients previously treated with CPAP or urulo palato pharyhgo plast for snoring or OSA, presence of upper airway disorders, a history and physical examination compatible with cardiopulmonary disease, the presence of airway obstruction (forced expiratory volume in 1 s (FEV_1_)/forced vital capacity (FVC)) less than 80 % of the predicted value, signs of pulmonary hyperinflation on pulmonary function tests, and curvilinear expiratory flow-volume curves. Subjects with evidence of neuromuscular diseases (such as myasthenia gravis, hypokalemia, or Guillian-Barre syndrome) were also excluded.

### Ethics approval

The study was approved by Ethical Committee of the People’s Hospital of Xinjiang Uygur Autonomous Region and informed consent was obtained from all participants.

### Study design

Based on overnight polysomnography (PSG), 114 male and 42 female subjects with AHI > 10/h were diagnosed with OSA and formed the OSA group. 8 males and 7 females were excluded for presenting with expiratory flow limitation as detected by pneumotachograph, the FEV_1_/FVC less than 80 % of the predicted value. Thus 106 middle-aged male (aged: 45 ± 10 years) and 35 female (aged: 50 ± 9 years) subjects with OSA finally remained as the OSA group.

Ninety-three male and forty-one female subjects who fulfilled the same inclusion and exclusion criteria, were identified without OSA as AHI ≤ 10/h on their PSG results and formed the non-OSA group. Twelve (6 males, 6 females) subjects without OSA were excluded due to presenting with expiratory flow limitation. Finally, 87 male and 35 female subjects were distributed into the non-OSA group.

### Measurement of lung function and volumes

All participants underwent standard spirometry and lung volume determinations, in line with American thoracic society/European respiratory society guidelines [24]. Maximal flow-volume loops was conducted for each subject with sitting position using MasterScreen pneumotachograph (Jaeger/Care Fusion, Germany). For each pulmonary function test, three maximal flow-volume loops were taken to determine FVC and FEV_1_; the largest one was retained to calculate the ratio of FEV_1_ to FVC (FEV_1_/FVC). Peak expiratory flow, maximum expiratory flow at 75 % (MEF_75%_), at 50 % (MEF_50%_), and at 25 % (MEF_25%_) of FVC were also measured. Static lung volumes were determined after spirometry using a MasterScreen body plethysmograph (Jaeger/Care-Fusion, Germany) while the subject was sitting in a sealed box. Thoracic gas volume at FRC level was measured while subjects made gentle pants against the shutter at a rate of <1/sec. ERV, inspiratory capacity and vital capacity were measured during the same maneuver. The mean of three technically acceptable FRC measurements was used to calculate total lung capacity (TLC) as FRC + inspiratory capacity and residual volume as TLC − vital capacity.

### Sleep studies

All participants underwent an overnight laboratory PSG including electroencephalography, electrooculogram, electromyogram of the chin, electrocardiogram, and recording of snoring and body position, respiratory efforts were detected with ribcage and abdominal piezo belts, oxygen saturation using pulse oximetry, according to the American Academy of Sleep Medicine guidelines [25]: airflow was monitored by a nasal pressure transducer, supplemented by an oro-nasal thermal sensor. A nasal pressure drop ≥30 % of baseline associated with ≥4 % desaturation and a duration ≥10 s were scored as hypopnea; At least 10 s drop on a thermistor peak signal excursion of ≥90 % from baseline and absence of airflow on a nasal pressure transducer were scored as apnea. In at least 90 % of the events’ duration met the amplitude reduction criteria. Severity of OSA was expressed as the total number of apneas plus hypopneas per hour of sleep (AHI).

### Measurement of mechanical properties of the respiratory system

IOS measurements and quality control were conducted in line with European respiratory society recommendations using Masterscreen IOS (Jaeger/Care-Fusion, Germany) [26]. IOS measurement was performed in each subject sitting in a neutral posture with cheek support while wearing noseclips. Subjects were asked to tightly seal their lips around the mouthpiece and to breathe quietly at FRC level. Continuous rectangular electrical impulse signals were superimposed on the airway via a mouthpiece after stable spontaneous volume and airflow were confirmed and a minimum of 3 consecutive measurements of >30 s were taken. As IOS measures we used respiratory system impedance (Zrs) at 5Hz (Zrs5), mean whole-breath values of Rrs and reactance (Xrs) between 5Hz and 35Hz in 5Hz increments (R5–R35 and X5–X35, respectively) and resonant frequency (Fres). IOS measurements were performed by two experienced technicians who were blinded to the study groupings. Measurements with artifacts, such as irregular breathing, hyperventilation, leakages or swallowing, were discarded. IOS can evaluate Rrs and Xrs at various oscillatory frequencies that are automatically calculated with computer software that uses fast Fourier transform analysis to determine Raw in extrathoracic and intrathoracic airways as well as the elastic properties of lung and chest wall. The Zrs encompasses all forces that hinder air flow into and out of the lung, and includes the resistance, elastance, and inertia of the system. The Rrs is a real part of Zrs, in phase with flow, which reflects energy dissipation due to resistive losses. Rrs is the sum of the extrathoracic airway, intrathoracic airway, and chest wall resistance, all arranged in series. In general, lower frequency data reflect the more peripheral regions of the lung, while higher frequency data are most representative of the central or proximal airways [20, 22, 26]. The Xrs is an imaginary component of Zrs that comprises out-of-phase lagging flow, which is elastance, and out-of-phase leading flow, which is inertia. Both of these components reflect energy storage. Theoretically, lung elasticity properties are reflected in the lower oscillatory-frequencies, while inertial properties are reflected dominantly in the higher oscillatory-frequencies [22, 26]. Rrs measured at lower frequencies (from 5 to 15 Hz) on IOS enabled us to obtain the total Rrs (Rrs at 0 Hz; R0) using the linear regression model R(f) = R0 + S × f, where f represents the frequency, S is the slope of the linear relationship of resistance versus frequency, R0 is equivalent to zero-order frequency resistance, namely the intercept). Grs was calculated as the reciprocal of R0 and sGrs was obtained as the ratio of Grs over FRC. In addition; the value of Zrs at 5Hz (Zrs5) yield by IOS is believed to be equivalent to R0, respiratory conductance was also calculated as the reciprocal of Zrs5 and expressed as Gz, the ratio of Gz over FRC as sGz.

### Statistical analyses

All data were expressed as mean ± standard deviation. Data for males and females were analyzed separately, because of differences between the genders in lung volume size and airway resistance, as well as the prevalence of OSA which are all well documented. Intergroup comparison was made using Independent-Samples *t*-test for variables showing normal distribution and homoscedasticity; otherwise, the Mann-Wilcoxon-test was used. Correlations among variables were determined using the least-square linear regression method. Multiple stepwise regression analysis was performed to assess relations between severity of OSA and lung volumes, respiratory mechanical properties and anthropometry. Statistical analysis was performed using SPSS (version 19.0, IBM Corp., Armonk, NY, USA). *p*-values < 0.05 were considered significant.

## Results

### Baseline characteristics

A total of 263 subjects were finally included in the study analysis. Of these, 68 males and 31 females with a BMI of 24–27 kg/m^2^ were classed as pre-obese; 136 males and 48 females had a BMI of 27.1–40 kg/m^2^ were classified as minimal and moderately obese; 3 males and 4 females were morbidly obese and had a BMI over 40 kg/m^2.^ Baseline data of anthropometric, pulmonary function, lung volumes and PSG results for all subjects are given in Table 1. There were significant reductions in absolute value of FRC and ERV in the OSA group compared to the non-OSA group among both genders. To further confirm that decreased lung volume was independent from BMI association with the severity of OSA as defined by AHI, multiple stepwise regression analysis was required to adjust for BMI or weight for males because weight and BMI for males were significantly greater in the OSA group than in the non-OSA group. The multiple stepwise regression analysis was performed with AHI as a dependent variable and the anthropometric data (age, height, weight, and BMI), lung volumes (TLC, residual volume, inspiratory capacity, and vital capacity) and ERV or FRC as independent variables. FRC and ERV were not entered into the regression model simultaneously as explanatory variables because they were highly interdependent with each other, the correlation coefficient was for males: r^2^ = 0.611, *p* < 0.001; for females: r^2^ = 0.649, *p* < 0.001 (Additional file 1: Figure S1). The analysis results are summarized in Table 2. The results revealed that a significantly reduction in FRC with a drop in ERV was independent from BMI associated, to a lesser extent, with the severity of OSA. This was true in females, significant reduced of FRC and ERV were found in the OSA group compared with the non-OSA group; Those lung volume were significantly correlated negatively to the severity of OSA, the spearman correlation coefficient between FRC or ERV and AHI was (r = −0.326, *p =* 0.006; r = −0.303, *p* = 0.011, respectively) and there were no significant differences in terms of weight and BMI, and other anthropometric data were found between the two groups

**Table 1:** Anthropometric, spirometric, lung volumes and nocturnal PSG data in the OSA group and non-OSA group for men and women

Variables	Men (*n* = 193)			Women (*n* = 70)		
	Non-OSA group (*n* = 87)	OSA group (*n* = 106)	*p-*Value	Non-OSA group (*n* = 35)	OSA group (*n* = 35)	*p*-Value
Age, years	44 ± 8	45 ± 10	0.605	47 ± 9	50 ± 9	0.091
Height, cm	172 ± 5.5	171 ± 6.3	0.269	158 ± 5.1	157 ± 6.0	0.266
Weight, kg	82 ± 10.4	90 ± 14.8	<0.001	74 ± 12.3	75 ± 13.5	0.698
BMI, kg/m^2^	28.0 ± 3.0	30.5 ± 4.2	<0.001	29.7 ± 4.9	30.7 ± 5.1	0.388
FVC, L	4.19 ± 0.50	4.06 ± 0.62	0.117	3.07 ± 0.49	2.83 ± 0.61	0.075
FEV_1_, L	3.36 ± 0.44	3.28 ± 0.53	0.188	2.47 ± 0.42	2.31 ± 0.47	0.125
FEV1/FVC, (% pred)	100 ± 6.0	101 ± 6.4	0.285	101 ± 7.4	103 ± 6.9	0.231
PEF, L/s	8.36 ± 1.33	8.12 ± 1.60	0.266	6.02 ± 1.10	5.78 ± 0.97	0.350
MEF_75_, L/s	7.16 ± 1.38	6.86 ± 1.40	0.132	5.39 ± 1.27	5.31 ± 1.15	0.785
MEF_50_, L/s	4.02 ± 0.96	3.80 ± 1.13	0.158	3.29 ± 1.13	3.04 ± 0.84	0.300
MEF_25_, L/s	1.43 ± 0.52	1.35 ± 0.56	0.138	1.12 ± 0.61	0.98 ± 0.40	0.275
MEF_25–75_, L/s	3.30 ± 0.87	3.13 ± 0.98	0.325	2.59 ± 0.97	2.40 ± 0.71	0.355
ERV, L	1.56 ± 0.55	1.26 ± 0.53	<0.001	1.09 ± 0.53	0.83 ± 0.40	0.022
FRC, L	3.73 ± 0.57	3.40 ± 0.62	<0.001	2.87 ± 0.57	2.60 ± 0.47	0.037
TLC, L	6.24 ± 0.61	6.08 ± 0.74	0.103	4.70 ± 0.48	4.50 ± 0.62	0.140
RV, L	2.17 ± 0.38	2.15 ± 0.41	0.740	1.78 ± 0.32	1.77 ± 0.32	0.953
IC, L	2.51 ± 0.55	2.68 ± 0.56	0.041	1.83 ± 0.39	1.90 ± 0.45	0.494
VC, L	4.07 ± 0.56	3.93 ± 0.64	0.124	2.92 ± 0.45	2.73 ± 0.53	0.102
ERV/TLC, %	25.0 ± 8.2	20.5 ± 7.8	<0.001	23.0 ± 9.4	18.4 ± 8.4	0.035
FRC/TLC, %	59.8 ± 7.5	55.9 ± 7.4	<0.001	61.0 ± 8.0	57.2 ± 7.8	0.046
RV/TLC, %	34.8 ± 5.6	35.5 ± 5.8	0.425	37.9 ± 6.3	39.5 ± 6.6	0.275
I C/TLC, %	40.2 ± 7.5	44.0 ± 7.6	0.001	39.1 ± 7.9	42.1 ± 8.0	0.121
VC/TLC, %	65.6 ± 5.6	64.6 ± 5.7	0.501	62.2 ± 6.3	60.5 ± 6.6	0.274
AI, events/h	0.2(0–4.4)	20.0(0–101.7)	<0.001†	0(0–2.7)	2.6(0–57.6)	<0.001†
HI, events/h	2.4(0–9.2)	9.7(0.1–40.9)	<0.001†	2.7(0–6.5)	20.0(2.8–86)	<0.001†
AHI, events/h	3.6(0–10)	30.8(10.4–102.1)	<0.001†	3.2(0–6.5)	23.6(5.4–86.2)	<0.001†

*Abbreviations*: *BMI* body mass index, *FVC* forced vital capacity, *FEV*_*1*_ forced expiratory volume in 1 s, *FEV*_*1*_ */FVC(%, pred)* ratio of FEV_1_ to FVC (ratio of the value to the predicted FEV1/FVC), *PEF* peak expiratory flow, *MEF*_*75,50,25*_ maximal expiratory flow at 75 %, 50 %, and 25 % of FVC, *ERV* expiratory reserve volume, *FRC* functional residual capacity, *RV* residual volume, *TLC* total lung capacity, *IC* inspiratory capacity, *VC* vital capacity, *ERV/TLC* ratio of ERV to TLC, *AI* apnea index, *HI* hypopnea index, *AHI* apnea + hypopnea index. Normally distributed data are expressed as mean ± SD; AI, HI and AHI are expressed as median (range). Comparison was made between the groups using the Independent-Samples *t*-test or *t’*-test, when data for variables showed a normal distribution and homogeneity or inhomogeneity of variances, otherwise, Wilcoxon test was used. A *p*-value below 0.05 was considered to be significant. † represents Mann–Whitney-Wilcoxon test

**Table 2:** Relationship between lung volumes and AHI after adjustment for BMI by multiple regression analysis in males

Model	Step	Explanatory variable	Standardized coefficients	*t*	*p*	Partial correlation	Collinearity statistics	
		Included variables	Beta				Tolerance	VIF
1	First	(Constant)		−4.830	<0.001			
		BMI	0.441	6.782	<0.001	0.441	1.000	1.000
2	Second	(Constant)		−3.271	0.001			
		BMI	0.413	6.297	<0.001	0.416	0.963	1.039
		ERV	−0.143	−2.188	0.030	−0.157	0.963	1.039
3		(Constant)		−1.943	0.054			
		BMI	0.408	6.156	<0.001	0.408	0.943	1.060
		FRC	−0.136	−2.043	0.042	−0.147	0.943	1.060
		Excluded variables						
A	First	age	0.042	0.615	0.539	0.045	0.921	1.086
		height	0.026	0.402	0.688	0.029	0.998	1.002
		weight	0.102	0.716	0.475	0.052	0.207	4.830
		TLC	−0.077	−1.183	0.283	−0.086	1.000	1.000
		RV	0.000	−0.008	0.993	0.000	0.991	1.009
		IC	0.045	0.679	0.498	0.049	0.946	1.057
		VC	−0.085	−1.318	0.189	−0.095	0.999	1.001

Dependent variable: apnea + hypopnea index (AHI). Independent variables: age, height, weight, body mass index (BMI) and total lung capacity (TLC), inspiratory capacity (IC), vital capacity (VC), residual volume (RV), expiratory reserve volume (ERV) or functional residual capacity (FRC). Models 1 and 2 derived from ERV; Models 1 and 3 derived from FRC combined with other independent variables. Variables shown in Model A were excluded variables and were derived from the first of the regression analysis with AHI as dependent and ERV or FRC combined with other independent variables

### Respiratory system mechanical properties

The mechanical properties of the respiratory system for both genders and comparison between the OSA and non-OSA groups are shown in Table 3. There were 13 male subjects who failed to arrange IOS measurements. The values of Zrs5 and Rrs at all frequencies for males were significantly higher in the OSA group than in the non-OSA group, while Xrs at 5–20 Hz were significantly lower. Among female subjects, there were an increasing trend in Rrs at all frequencies in the OSA group compared with the non-OSA group, but, significant differences were found only in Zrs5 and R5 between the two groups, although Xrs from 5 to 25 Hz were significantly lower.

**Table 3:** Comparison of the mechanical properties of respiratory measurement using IOS for men and women

Parameters	Men (*n* = 180)			Women (*n* = 70)		
	Non-OSA group (*n* = 83)	OSA group (*n* = 97)	*p-*Value	Non-OSA group (*n* = 35)	OSA group (*n* = 35)	*p*-Value
Zrs5, (kPa/L/s)	0.37 ± 0.11	0.45 ± 0.13	<0.001	0.53 ± 0.14	0.61 ± 0.18	0.033
R5, (kPa/L/s)	0.35 ± 0.10	0.43 ± 0.12	<0.001	0.50 ± 0.13	0.57 ± 0.15	0.041
R10,(kPa/L/s)	0.32 ± 0.09	0.38 ± 0.09	<0.001	0.44 ± 0.11	0.47 ± 0.11	0.140
R15,(kPa/L/s)	0.30 ± 0.07	0.34 ± 0.08	<0.001	0.40 ± 0.10	0.42 ± 0.09	0.242
R20,(kPa/L/s)	0.30 ± 0.07	0.35 ± 0.08	<0.001	0.40 ± 0.09	0.42 ± 0.10	0.350
R25,(kPa/L/s)	0.30 ± 0.08	0.36 ± 0.09	<0.001	0.41 ± 0.08	0.42 ± 0.09	0.434
R35,(kPa/L/s)	0.33 ± 0.07	0.39 ± 0.09	<0.001	0.43 ± 0.08	0.45 ± 0.09	0.317
Grs, (L/s/kPa)	2.78 ± 0.64	2.26 ± 0.50	<0.001	1.92 ± 0.45	1.61 ± 0.33	0.002
sGrs, (s^−1^ · kPa^−1^)	0.76 ± 0.19	0.68 ± 0.17	0.007	0.68 ± 0.17	0.63 ± 0.17	0.171
Gz, (L/s/kPa)	2.94 ± 0.75	2.40 ± 0.63	<0.001	2.04 ± 0.55	1.76 ± 0.44	0.021
sGz, (s^−1^ · kPa^−1^)	0.80 ± 0.22	0.72 ± 0.19	0.031	0.72 ± 0.19	0.69 ± 0.21	0.363
X5,(kPa/L/s)	−0.08 ± 0.05	−0.13 ± 0.08	<0.001	−0.15 ± 0.07	−0.22 ± 0.13	0.008
X10,(kPa/L/s)	−0.03 ± 0.04	−0.05 ± 0.06	<0.001	−0.07 ± 0.06	−0.12 ± 0.09	0.030
X15,(kPa/L/s)	0.00 ± 0.04	−0.02 ± 0.05	<0.001	−0.04 ± 0.06	−0.08 ± 0.08	0.033
X20,(kPa/L/s)	0.03 ± 0.03	0.02 ± 0.04	0.002	0.03 ± 0.18	−0.03 ± 0.06	0.029
X25,(kPa/L/s)	0.07 ± 0.03	0.06 ± 0.04	0.060	0.05 ± 0.04	0.02 ± 0.05	0.027
X35,(kPa/L/s)	0.12 ± 0.04	0.11 ± 0.04	0.646	0.09 ± 0.04	0.09 ± 0.04	0.911
Fres,(Hz)	14.4 ± 4.7	17.7 ± 6.0	<0.001	19.4 ± 7.4	23.5 ± 9.3	0.081

*Abbreviations*: *Zrs5* respiratory system impedance at 5Hz (oscillatory frequency), *R5 ~ R35* respiratory system resistance at 5, 10, 15, 20, 25, 35 oscillatory frequencies, *X5 ~ X35* respiratory system reactance at 5, 10, 15, 20, 25, 35Hz, *Grs* respiratory conductance, reciprocal of R0 (total respiratory resistance, namely the resistance origin at the point of zero frequency), *sGrs* the specific Grs (the ratio of Grs to actual value of FRC), *Gz* reciprocal of Zrs5, *sGz* the specific Gz (the ratio of Gz to actual value of FRC), *Fres* resonant frequency, integrated area of low-frequency reactance from zero line between X5 and resonant frequency; IOS parameters are expressed as mean ± SD. Comparison was made between the two group using Mann–Whitney-Wilcoxon test. A *p*-value below 0.05 was considered to exist significant difference

**Fig. 1:**
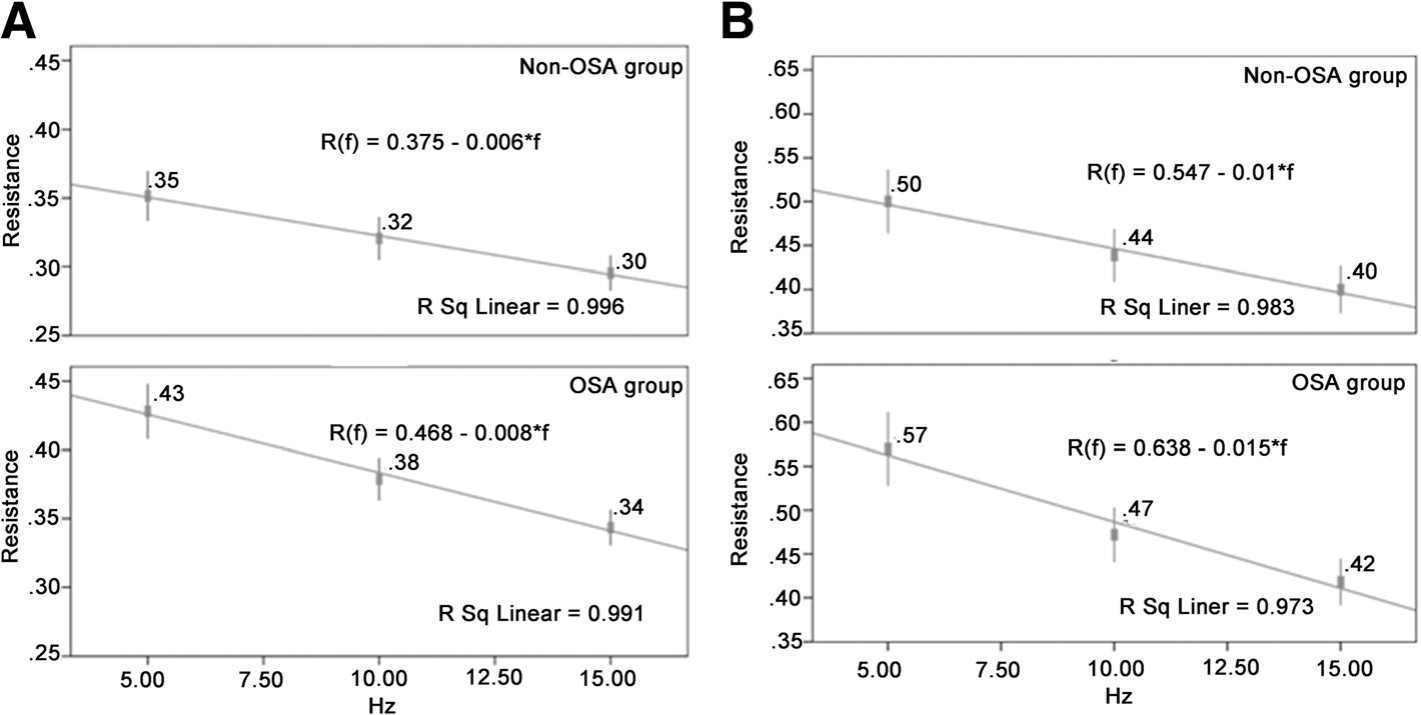
**a** Respiratory resistance (Rrs) from 5 to 15Hz subjected to linear regression analysis of resistance vs. frequency and resistance at the point of zero-frequency (R0) in males in the non-OSA and OSA groups. **b** Rrs from 5 to 15Hz subjected to linear regression analysis of resistance vs. frequency and resistance at R0 in females in the non-OSA and OSA groups

**Fig. 2:**
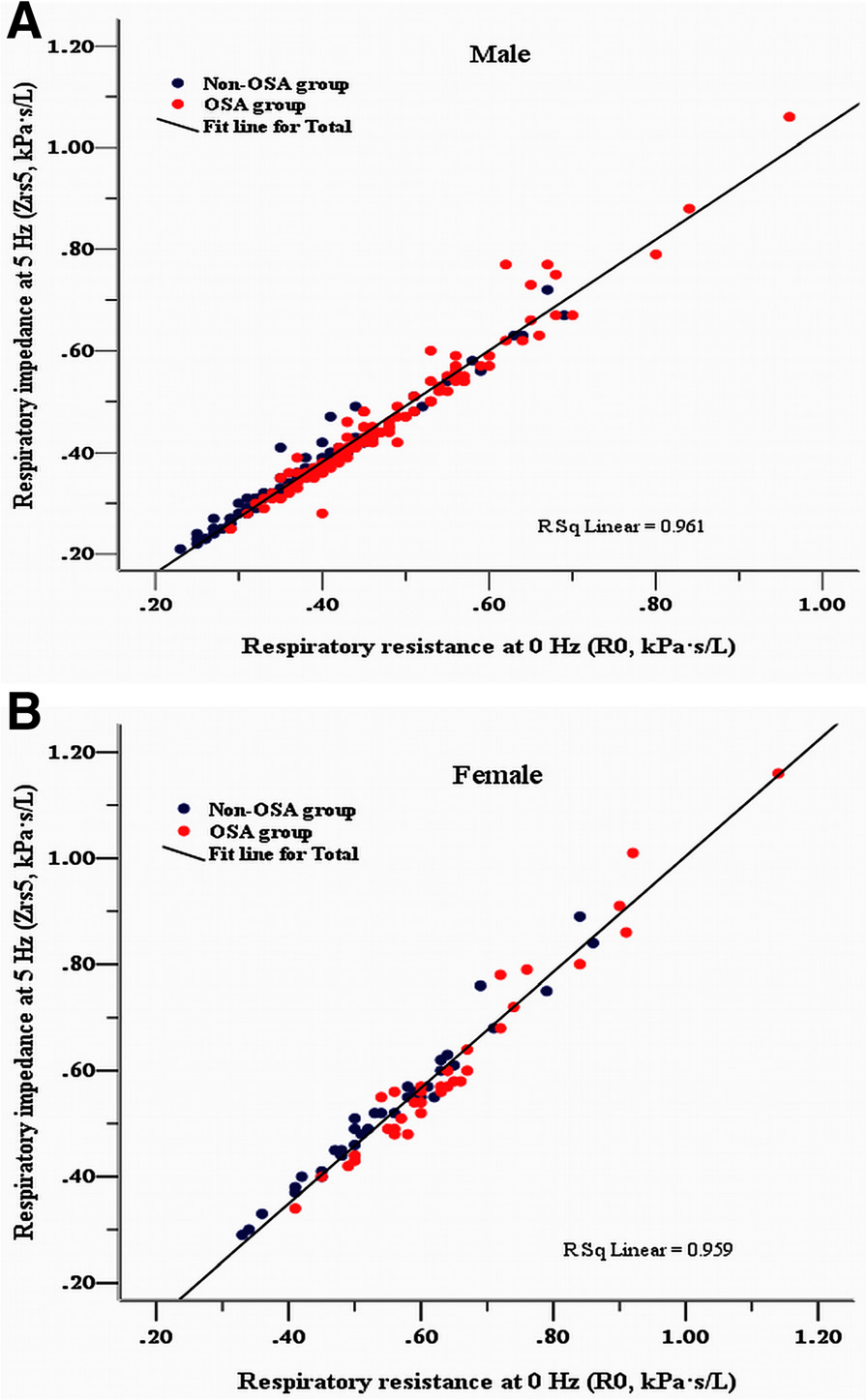
**a** and **b** Correlation between respiratory system impedance at 5 oscillatory frequency (Zrs5) and back extrapolation of zero-frequency resistance (R0) in males and females (**a** and **b**)

Based on the values of Rrs at lower frequencies (4–16 Hz) measured by FOT were characterized by negative frequency dependence (Rrs increased linearly with a decrease of oscillatory frequency) in upper airway obstruction patients and in obese subjects with and without OSA. Similarly, the characteristics mentioned above were observed in this study population; thus, the total Rrs—namely, Rrs at zero-frequency (R0) can be back-extrapolated by applying linear regression analysis of mean whole-breath resistance vs frequency over the 5–15 Hz on IOS. The linear regression model and equations are shown in Fig. 1a and b. R0 was calculated separately, according to each subject in each different condition by the linear model and equation (Fig. 1a and b); then the reciprocal of R0—namely Grs was obtained and The sGrs was also calculated as the ratio of Grs over FRC. Significant decreases in Grs and sGrs, and a significant increase in R0 were found in the OSA group compared with the non-OSA group for males. These results were also found among female subjects between the two groups, except that sGrs was not significantly different between the OSA and the non-OSA group (0.63 ± 0.17 vs. 0.68 ± 0.17, *p =* 0.171). The Zrs5 yield by IOS for male and female subjects in the OSA and the non-OSA group were found to be approximately equal to R0 and highly correlated with R0 in all subjects (Fig. 2a and b); therefore, results of the reciprocal of Zrs5 (Gz) and sGz (the ratio of Gz over FRC) were found similar to the results of Grs and sGrs mentioned previously.

### Relationships between IOS measurements and severity of OSA

**Fig. 3:**
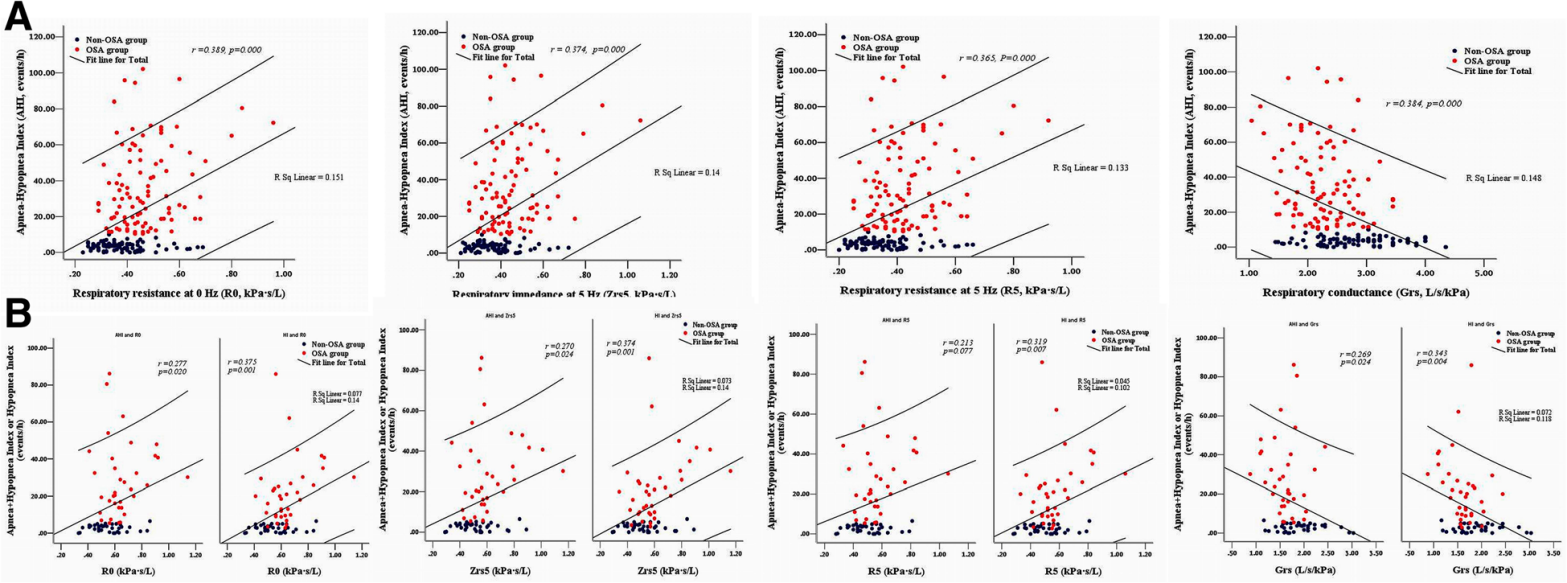
**a** Correlation between severity of obstructive sleep apnea (OSA) as defined by Apnea-Hypopnea Index (AHI) and zero-frequency resistance (R0), Zrs5, R5, and Grs in males. **b** Correlation between severity of obstructive sleep apnea (OSA) as defined by Apnea-Hypopnea Index(AHI) or Hypopnea Index(HI) and zero-frequency resistance (R0), Zrs5, R5, and Grs in females

Figure 3a and b show that significant increases in R0, Zrs5 and R5 and a decrease in Grs were found with increasing severity of OSA among male subjects and those parameters were moderately correlated with AHI. Similarly relationships were also found in females between parameters of R0, Zrs5, R5, and Grs and hypopnea index. Because those parameters were found to be weakly associated with AHI, further analysis was required and we found that female subjects who mainly manifested hypopnea in their PSG showed more hypopnea than apnea. There was no significant correlation between sGrs and severity of OSA as defined by AHI or hypopnea index in female subjects, and only a weak correlation between those variables in males (*r* = −0.198, R^2^ = 0.039, *p* = 0.008).

We examined the relationships between reactance (Xrs) and Rrs, because airway resistance is not only related to lung volume, but also depends on lung elasticity recoil pressure. In addition, lung compliances would be low or its inverse lung elasticity would be high due to breathing at abnormally low lung volume. Xrs is made up of out-of-phase lagging flow and leading flow, exhibiting numerically a negative value that reflects the sum elastance or its inverse compliance of the lung and chest wall. Lung elasticity properties are thought to be reflected in low–oscillatory frequencies, while inertial properties are dominant in high–oscillatory frequencies and are believed to reflect chest wall compliance or elastance, so the Xrs values in lower oscillatory frequencies that are more negative indicate increased lung elasticity or reduced the compliance. As we expected, Xrs at 5 Hz (Xrs5) for male and female subjects were found to be highly correlated with Zrs5, R0 and R5, the Spearman correlation coefficients are summarized in Table 4. There was also a strong correlation between Xrs5 and severity of OSA in male and female subjects. To determine whether alterations in Xrs for male and female subjects were independently associated with the severity of OSA multiple stepwise regression analysis was performed with AHI as a dependent variable and the anthropometric data (age, height, weight, and BMI) and mechanical properties of the respiratory yielding by IOS (Zrs5, R0, Grs, R5, Xrs at 5, 10, 15 Hz) as independent variables. The analysis results are summarized in Table 5. The Xrs5 was found to be highly independently correlated with AHI, and an even more highly independent correlation between Xrs5 and HI was found in female subjects.

**Table 4:** Spearman correlation coefficient between respiratory reactance at 5 Hz (Xrs5) and Zrs5, R0, and R5 for male and female subjects

Parameter	Male (n = 180)	*p*	Female (n = 70)	*p*
	Xrs5		Xrs5	
Respiratory impedance at 5 Hz (Zrs5)	−0.638	<0.001	−0.725	<0.001
Respiratory resistance at 0 Hz (R0)	−0.590	<0.001	−0.644	<0.001
Respiratory resistance at 5 Hz (R5)	−0.577	<0.001	−0.637	<0.001

**Table 5:** Multiple stepwise regression analysis of the association of reactance measured at 5Hz with the severity of OSA

Model		Unstandardized Coefficients	Standardized Coefficients	*t*	*P*	95 % Confidence Interval for B		Correlation coefficients		
		B	Beta			Lower Bound	Upper Bound	Zero-order	Partial	Part
1	Constant	2.623		0.916	0.361	−3.028	8.274			
	X5	−173.294	−0.504	−7.784	<0.001	−217.288	−129.361	−0.504	−0.504	−0.504
2	Constant	−40.982		−4.404	<0.001	−59.344	−22.619			
	X5	−144.800	−0.421	−6.659	<0.001	−187.714	−101.887	−0.504	−0.448	−0.406
	weight	0.584	0.310	4.896	<0.001	0.327	0.786	0.422	0.345	0.298
3	Constant	0.225		0.056	0.955	−7.775	8.225			
	X5	−85.332	−0.482	−4.539	<0.001	−122.847	−47.819	−0.482	−0.482	−0.482
4	Constant	−25.415		−2.061	0.043	−50.035	0.796			
	X5	−74.946	−0.424	−3.966	<0.001	−112.688	−37.223	−0.482	−0.436	−0.410
	BMI	0.912	0.234	2.191	0.032	0.081	1.744	0.340	0.259	0.227
5	Constant	−3.143		−1.046	0.299	−9.141	2.855			
	X5	−84.601	−0.589	−6.002	<0.001	−112.726	−56.476	−0.589	−0.589	−0.589
6	Constant	−23.130		−2.059	0.015	−41.536	−4.726			
	X5	−76.504	−0.532	−5.415	<0.001	−104.704	−48.305	−0.589	−0.552	−0.515
	BMI	0.711	0.225	2.258	0.025	0.090	1.333	0.358	0.269	0.217

Independent variables: Zrs5, Gz, sGz, Grs, sGrs, R5, X5, X10, X15; Dependent Variable: AHI or HI. Model 1, 2 derived from male subject data by performing stepwise regression analysis; model 3, 4 derived from female subject data; model 5, 6 derived from female subject data with HI as a dependent variable

*Abbreviations*: *BMI* body mass index, *X5* respiratory system reactance at 5Hz, *AHI* apnea + hypopnea index, *HI* hypopnea index, *Zrs5* respiratory system impedance at 5Hz (oscillatory frequency), *R5* respiratory system resistance at 5 oscillatory frequencies, *Grs* respiratory conductance, reciprocal of R0 (total respiratory resistance, namely the resistance origin at the point of zero frequency), *sGrs* the specific Grs (the ratio of Grs to actual value of FRC), *Gz* reciprocal of Zrs5, *sGz* the specific Gz (the ratio of Gz to actual value of FRC)

### Relationships between lung volume and respiratory mechanical properties

**Fig. 4:**
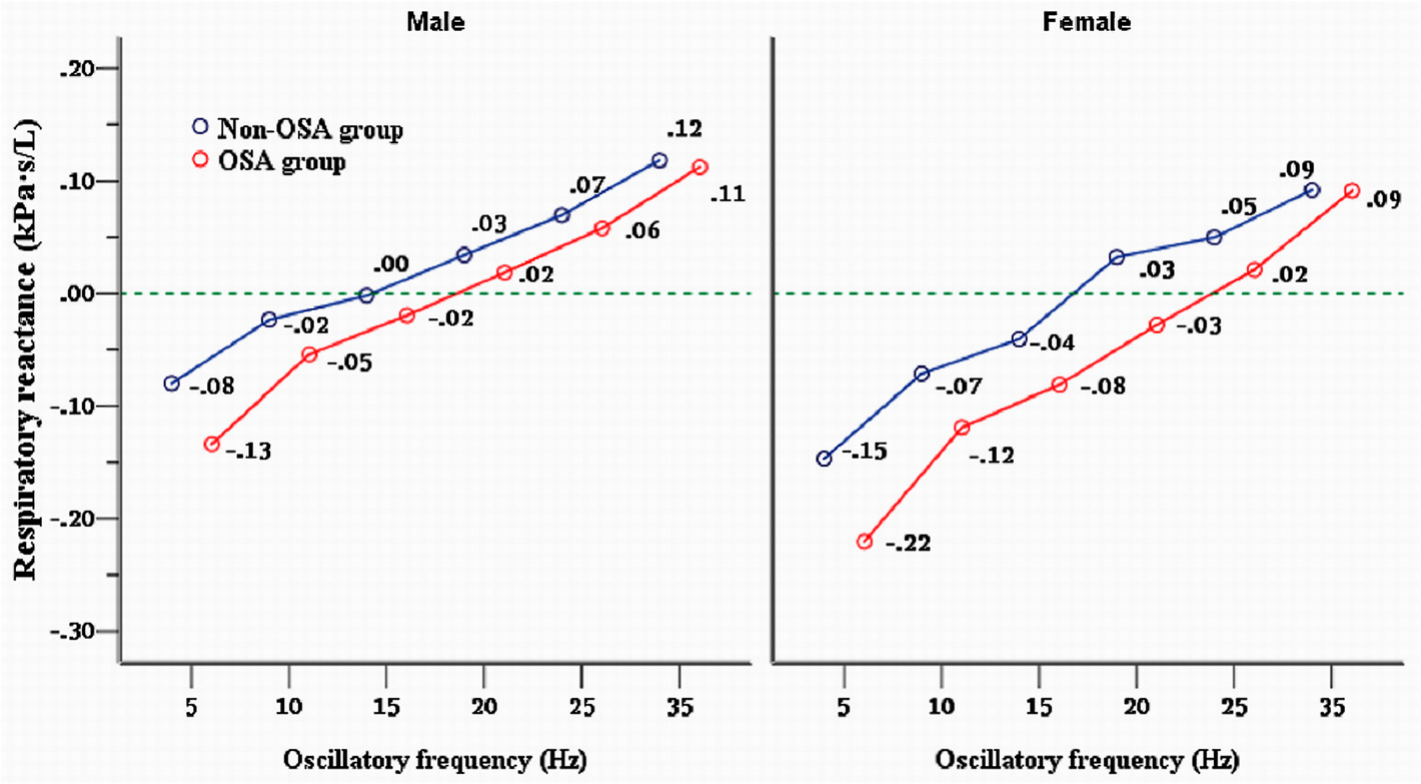
Respiratory reactance at different oscillatory frequency measured by IOS for males and females subjects in the non-OSA and OSA groups. Solid line between the reactance at various oscillatory frequency represent linear interrelationship were plotted using the least squares method

At rest, under static conditions, FRC is dependent on the balance of lung elasticity inward recoil forces and chest wall outward recoil forces. Thus, FRC is very sensitive to alterations in compliance of the lungs and chest wall. Increases in elastance or its inverse compliance (the reciprocal of elastance) reduction in lung and chest wall are related to a decrease in FRC. To determine whether the Xrs measured by IOS was sensitive to detect FRC alterations in male and female subjects with and without OSA, stepwise-regression analysis was performed with FRC as a dependent variable, height, BMI, Xrs at all frequencies, and AHI were also included in the regression equation as independent variables. The analysis results are summarized in Table 6. The results revealed that the parameter of Xrs5 alone could account for, as much as 32 % of the variation in FRC for both genders. In males, BMI as independent variable together Xrs5 contributed to increase the predictive value of FRC slightly; That increase was also found when we included AHI instead of BMI in the equation, because they had similar coefficients with FRC. The correlation coefficient between FRC and BMI or AHI are shown in Table 6, and revealed that obesity defined by BMI negatively impacts FRC, similar to AHI effecting FRC. Our results also revealed that FRC was significantly associated with low-oscillatory frequency reactance, since we found the absolute value of Xrs from 5Hz to 15 Hz was significantly negatively correlated with FRC in both genders, while there was no relationship between high-frequency Xrs (from 25 to 35Hz) and FRC, despite high interdependent relationships existing among Xrs at different frequencies Fig. 4.

**Table 6:** Correlation between FRC and reactance at all frequencies derived from stepwise regression analysis in males and females

Model	Explanatory variable	Standardized coefficients		*t*		*p*		Partial correlation		Co-linearity statistics	
	Included variables	Beta Male	Beta Female	Male	Female	Male	Female	Male	Female	Tolerance Male	Tolerance Female
1	(Constant)			46.575	25.286	<0.001	<0.001				
	X5	0.320	0.326	4.501	2.842	<0.001	0.006	0.320	0.326	1.000	1.000
	Excluded variables										
A	X10	0.106	0.090	0.907	0.343	0.366	0.732	0.068	0.042	0.366	0.193
	X15	0.107	0.081	1.091	0.394	0.277	0.695	0.082	0.048	0.523	0.314
	X20	0.160	0.001	1.846	0.010	0.067	0.992	0.137	0.001	0.663	0.870
	X25	0.023	−0.089	0.290	−0.596	0.772	0.553	0.022	−0.073	0.784	0.597
	X35	0.005	−0.061	0.070	−0.478	0.945	0.634	0.005	−0.058	0.865	0.817
	AHI	−0.133	−0.121	−1.745	−1.025	0.083	0.309	−0.130	−0.124	0.853	0.937
	BMI	−0.111	−0.103	−1.351	−0.784	0.178	0.436	−0.101	−0.095	0.747	0.767

Dependent variable: functional residual capacity (FRC). Independent variables: respiratory reactance at 5, 10, 15, 20, 25, 35 Hz (X5 ~ X35), apnea + hypopnea index (AHI) and body mass index (BMI). Variables showed in Model A were excluded variables and was derived from the regression analysis

## Discussion

This study explored the effect of OSA on lung volumes and mechanical properties of the respiratory system in pre-obese/obese subjects. The results showed that OSA impacts upon lung elasticity properties, and they increased with OSA severity. There was also a suggested increased airflow resistance in the extrathoracic and peripheral airways and a decrease in lung volume, in terms of FRC and ERV in obese OSA. The unique and novel aspects of this study were to measure Zrs5 in the morning after an overnight sleep study, and Rrs and Xrs were also measured at different frequencies using IOS with a standardized method. Many previous studies have confirmed that lung function is little changed with class I and II obesity [27–28, 29, 30]. Some research suggests obesity is associated with a higher ratio of FEV_1_ to FVC [28, 30]. Meanwhile, there is general agreement that conventional pulmonary function tests are unhelpful for diagnosis of OSA [31]. In the present study, conducted on a large number of pre-obese/obese subjects with and without OSA, we were not surprised that there was no significant difference in FEV_1_/FVC between obese subjects with and without OSA, although the FEV_1_/FVC were slightly higher in the OSA group than non-OSA group.

Hoffstein *et al.* demonstrated that the cross-sectional area of the pharyngeal-airway decreases as lung volume decreases from FRC to residual volume, a phenomenon most pronounced in obese OSA patients [32, 33]. Several studies since then have associated lung volume with severity of OSA. Appelberg *et al.* reported ERV was significantly lower in subjects with OSA compared with non-snoring subjects and that ERV was independently correlated to apnea index and oxygen desaturation frequency [34]. Onal *et al.* found predicted FRC was significantly negatively correlated to AHI in OSA patients [15]. Zehra-Lancner *et al.* evaluated pulmonary function and lung volume in 170 obese snorers with or without OSA, and found all of patients had significant decreases in FRC, most markedly decreases in ERV, compared to predicted values [17]. In the present study, actual ERV significantly decreased with a drop of FRC in both genders of OSA patients compared with non-OSA subjects. However, decreases in those lung volume measurements have poor predicted values for AHI, we reasoned that size of lung volume *per se* may play a weak role in the mechanism of upper airway collapse. Our results also indicated that OSA had a negative impact on lung volume, similar to the impact of BMI on lung volume. Obesity is a common feature of OSA [35], and has been associated with decreased FRC with a drop in ERV [27–28] due to reduced compliance in the respiratory system. Zehra reported that lung volumes showed a marked decrease because of reduced chest wall compliance as the degree of obesity rose [16]. More recently, Behazin *et al.* and Pelosi *et al.* [36, 37] reported that obese subjects breathe at abnormally lower FRC than non-obese subjects due principally to reduced lung compliance rather than chest wall compliance. Our findings in this study appear to indicate that FRC and ERV were decreased more in pre-obese and obese patients with OSA than in those without OSA. The multiple regression analyses demonstrated that the lung volume decreases in obese men with OSA were independent of BMI and negatively correlated with AHI. This result was further confirmed by the female cohort; we classified the cohort according to the presence and absence of OSA, and found OSA group respiration at lower lung volumes than the non-OSA group and the two female groups had similar BMI. Recently, study animals were exposed to several hours of IRB, mimicking upper airway obstructed breathing, the researchers found that lung elasticity significantly increased in the IRB animals compared to the spontaneously breathing animals and found a downward shift of the pressure-volume-curve, suggesting that IRB leads to large negative swings in intrathoracic pressure [13]. Bijaoui reported that the onset of obstructed breathing was accompanied by an increased lower component of lung elasticity; simultaneously lung resistance increased when obstructed breathing existed in various sleep stages of OSA patients [38]. Xrs5 is numerically a negative value on IOS and known as lung elasticity properties or its inverse the compliance, so a more negative value indicates increased pulmonary elastance or reduced compliance (the reciprocal of elastance) we infer that this was a result of increased lung elasticity or its inverse, reduced compliance. Interestingly, in this study the Xrs5 was found to be strongly associated not only with the severity of OSA as defined by the AHI or HI, but also with the total airway resistance (R0 or Zrs5) as well as peripheral airways resistance (R5). This result suggested that with increasing severity of OSA pulmonary elastance might be markedly increased or its inverse—namely, the compliance was decreased, which in turn contributed to a significantly decreased FRC and marked increases of airflow resistance in both the upper airway and in peripheral airways in obese OSA patients than those who are solely obese; as it is well documented increased lung elastance or reduced the compliance is associated with low FRC as well as breathing at low lung volumes are related to increases resistance in the structures of upper airway and peripheral airways.

It has been established that a decreasing curvilinear relationship between FRC and total airway resistance or lung resistance in obesity [36, 39, 40]. This relationship between FRC and respiratory resistance on IOS was also found in our study population (Additional file 2: Figure S2. A, B). Van Noord *et al.* initially reported that values of Rrs at lower frequencies (4–16 Hz) measured by FOT are characterized by negative frequency dependence in patients with UA and bronchial obstruction; simultaneously they found that the Xrs values at lower frequencies showed a marked decrease (more negative) with decreased oscillatory frequency in upper airway obstruction patients [41]. Since Rrs increases linearly with a decrease of oscillatory frequency over lower frequencies as observed by Zerah *et al.* in obese subjects and the total airway resistance (R0) increased with level of obesity, this suggests that R0 increased in obese subjects resulting from the reduction in lung volume related to being overweight [16]. The researchers then demonstrated that with increasing severity of OSA in obese snorers, the R0 was a significantly increased and the reciprocal of R0, the Grs and the sGrs were significantly independent of BMI decreased with increasing AHI [17]. As result of the sGrs in those patients was found to be highly independently correlated with AHI and had a stronger predictive value for OSA [17, 18] the researchers suggest that upper airway and peripheral airway obstruction coexisted in obese OSA patients. In this study, we also found a significant increase in R0 and decrease in Grs with severity of OSA in both genders. However, we could not find any correlation between severity of OSA and sGrs in females, and only a weak correlation in males. We further analyzed our data in terms of ratio of Grs to normal predicted FRC, and found a stronger correlation between severity of OSA and sGrs, the result was similar to Zerah *et al.* in a previous study [17]. However, it can be speculated that the connection between OSA and sGrs would be overstated by this method. Small sGrs were obtained in our OSA-patients by dividing Grs with normal predicted FRC which has tendency to be bigger than the actual FRC, therefore, this yielded the small sGrs and a significant decrease of sGrs in OSA patients was found, the connection can be naturally exaggerated, although in fact OSA patients are breathing at low FRC.

A study clearly demonstrated breathing at low  lung volume  with a less lung compliance produces large swings in intrathoracic pressure in obese subjects and with which airways are prone to close or collapse on exhalation. Similarly, numerous studies have shown especially large swings in intrathoracic pressure exist when OSA occurs in OSA patients. Large swings in intrathoracic pressure means a high driving pressure or lung elasticity recoil pressure is generated at the end of inspiration or start of expiration, as well as a high negative pressure generated at the end of expiration or beginning of inspiration. Application of small sub-atmospheric pressure (from −3 to −10 cmH_2_O) at the start of expiration is defined as the negative expiratory pressure (NEP) technique, and is used for early detection of whether subjects have expiratory flow limitation (EFL) in their peripheral airways. However, The association between the degree of EFL induced by NEP and the severity of OSA has been well documented in OSA patients in the past twenty years, and it has been concluded that EFL caused by NEP are common in a population of OSA patients, which can be highly predictive the severity of OSA [42–44].

By putting such evidence together, it can be speculated that small additional NEP applied at the start of expiration for OSA patients, would further elevate intrathoracic driving pressure or lung elasticity recoil pressure [40, 42], if this already existed, consequently, higher driving pressure or lung elasticity recoil pressure can make alveoli and their opening (peripheral-airway) as well as the extrathoracic airway easily collapse and manifest through markedly increased airflow resistance in those structures. This phenomenon was also shown by many previous studies; for example elevating driving pressure by continuous negative airway pressure (CNAP) or positive extrathoracic pressure for healthy subjects, have always found significant increases in resistance in peripheral airways as well as upper airways that are simultaneously accompanied by slightly decreased lung volume [9, 40], which are remarkable in OSA patients [32, 33]. We believe, the converse would also apply, for example, decreasing driving pressure or lung elasticity recoil pressure by NETP or CPAP, improves upper airway collapsibility as well as in the intrathoracic-airway, and manifests as increased lung volume and markedly decreased in resistance in upper airway and intrathoracic airways [7–12]. A study has clearly demonstrated that the CNAP-induced increase in Rrs does not only result from a direct lung volume effect and strongly suggested the involvement of other factors that affect the caliber of intrathoracic and extrathoracic airways [40]. That was true, in fact, Rrs depends on lung elasticity recoil pressure [16], and lung resistance always increases with increase in lung elasticity [38, 37], in the current study we also found Rrs was significantly high correlated with Xrs5 in all study subjects. This evidence makes us conclude that Xrs5 could reflect the lung elasticity recoil pressure in this study. From such evidence, combined with our findings, we infer and conclude that upper airway obstruction together obesity negatively impacts mainly upon lung elasticity or lung compliance, which might be abnormally increased or the compliance decreased, respiration in a less lung compliance lead to increased lung elasticity recoil pressure on exhalation and contribute to a decrease in FRC, which, in turn, aggravate upper airway and intrathoracic airways obstruction or collapsibility and manifest through markedly increased resistance in upper airway and intrathoracic airways.

This study harbors some limitations. To explicate the effect of OSA on lung volumes and mechanical properties of respiratory, with the goal of eliminating a confounding effect of BMI differences on respiratory function tests result, initially, we carried out a case–control study. Although we found that the BMI matched in the female OSA and non-OSA subjects. This was not the case for the male groups. It is a challenge, in the moderately obese male population, to find subjects whose AHI < 5/h or even less than 10/h to match in terms of BMI. Therefore, there was a significant difference in BMI between the male groups. As this study was not intended to evaluate the relationship between BMI and OSA we then had to perform statistical analysis to account for these differences. Secondly, the nature of the study, as a cross-sectional observational one, limits the ability to ascertain the cause of the disease, and does not allow us to draw a causal relationship between respiratory system elastance or compliance and upper airway function in obese OSA patients, thus interventional animal studies are required to further confirm our findings.

## Conclusion

In conclusion, OSA has an impact on the elastic properties of the lung, which may increase with severity of OSA. Lung elasticity recoil pressure is increased in OSA patients due to increases of lung elasticity recoiling, which in turn, may take responsibility for decreased lung volume and increased airflow resistance in the upper and intrathoracic airways in OSA patients. This information might indicate new insights into pathogenesis of OSA; that narrowing of the upper airway and abnormally increased lung elasticity recoil pressure on exhalation may contribute to OSA.

## Additional files

Additional file 1: Figure S1. Relationships between functional residual capacity (FRC) and expiratory reserve volume (ERV) for the two study groups. **A:** Male subjects showed a close correlation between FRC and ERV. **B:** Female subjects also showed a close correlation between FRC and ERV. Additional file 2: Figure S2. Correlation between functional residual capacity (FRC) and respiratory resistance.

## Abbreviations

OSA: Obstructive sleep apnea
IOS: Impulse oscillation system
BMI: Body mass index
ERV: Expiratory reserve volume
FRC: Functional residual capacity
NEP: Negative extrathoracic pressure
AHI: Apnea-hypopnea index
Ttx: Traction on the trachea
EELV: End-expiratory lung volume
EFL: Expiratory flow limitation
IRB: Inspiratory resistive breathing
FOT: Forced-oscillation technique
Zrs: Respiratory system impedance
Zrs5: Zrs at 5Hz
Gaw: Airway conductance
Raw: Airway resistance
Rrs: Respiratory system resistance
Grs: Respiratory conductance
sGrs: Specific Grs
FEV1: Forced expiratory volume in 1 s
FVC: Forced vital capacity
Xrs: Reactance
Fres: Resonant frequency
Gz: Reciprocal of Zrs5
sGz: The ratio of Gz over FRC
