# Supplementary figures and images for: Impact of obstructive sleep apnea on lung volumes and mechanical properties of the respiratory system in overweight and obese individuals
Source: BMC Pulm Med. 2015 Jul 25;15:76. doi: 10.1186/s12890-015-0063-6 (PMC4513967; doi:10.1186/s12890-015-0063-6)

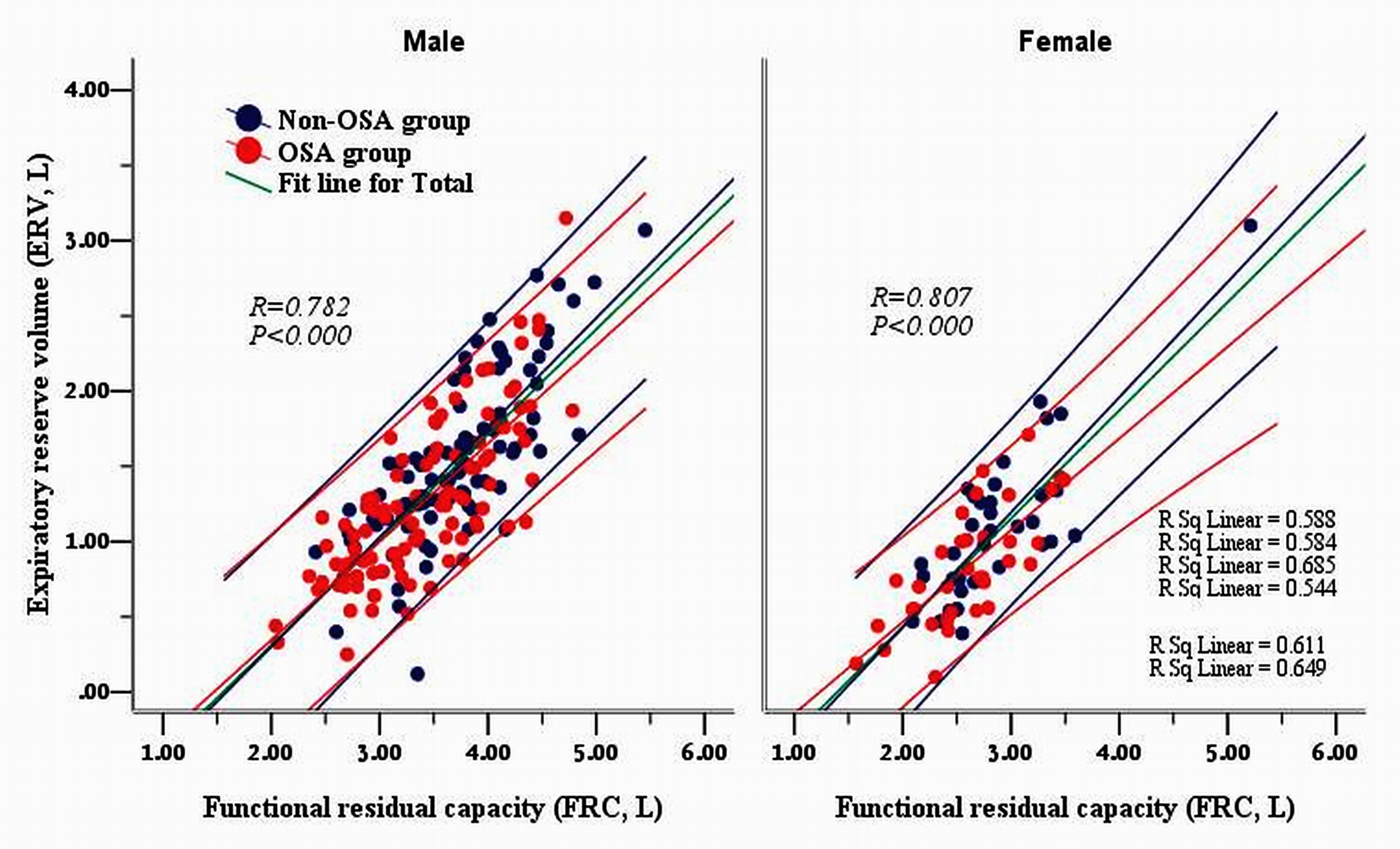

Supplement: Additional file 1: Figure S1. — Relationships between functional residual capacity (FRC) and expiratory reserve volume (ERV) for the two study groups. A: Male subjects showed a close correlation between FRC and ERV. B: Female subjects also showed a close correlation between FRC and ERV. [file 12890_2015_63_MOESM1_ESM.tiff]

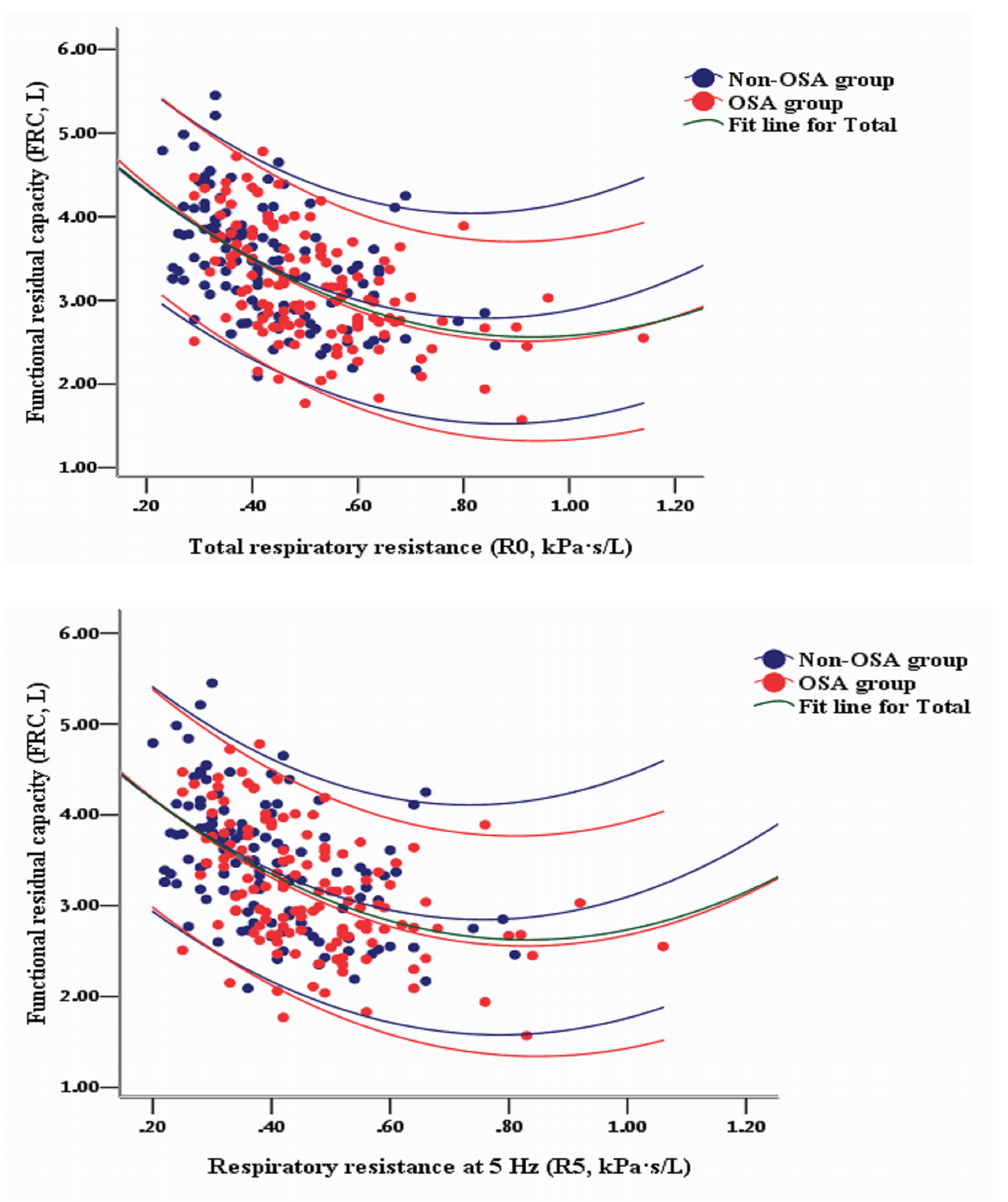

Supplement: Additional file 2: Figure S2. — Correlation between functional residual capacity (FRC) and respiratory resistance. [file 12890_2015_63_MOESM2_ESM.tiff]
